# Detection of Positive and Negative Pressure in a Double-Chamber Underwater Thruster

**DOI:** 10.3390/mi16050526

**Published:** 2025-04-29

**Authors:** Chong Cao, Chengchun Zhang, Chun Shen, Yasong Zhang, Wen Cheng, Zhengyang Wu, Luquan Ren

**Affiliations:** 1 Key Laboratory of Bionic Engineering (Ministry of Education), Jilin University, Changchun 130022, China; caocchina@163.com (C.C.);; 2 College of Automotive Engineering, Jilin University, Changchun 130022, China

**Keywords:** negative pressure, underwater thruster, pressure sensor

## Abstract

The aim of this paper is to develop a compact, rapid-response pressure sensor for underwater propulsion. Flexible pressure sensors are widely utilized in human–computer interactions and wearable electronic devices; however, manufacturing capacitive sensors that offer a broad pressure range and high sensitivity presents significant challenges. Inspired by the dermal papillary microstructure, a capacitive pressure sensor was prepared by infusing polydimethylsiloxane (PDMS) inside an anodic aluminum oxide (AAO) template and then demolding it. The resulting pressure sensor exhibits several key characteristics: high linearity in the range of −5.2 to 6.3 kPa, a comprehensive range for both positive and negative pressure sensing in air or water environments, a quick response time of 52 ms, a recovery time of 40 ms, and excellent stability. The sensor presented in this work is innovatively applied to detect underwater negative pressure, and it is employed for the swift detection of positive and negative pressure changes in underwater thrusters. This work highlights the promising potential of biomimetic flexible capacitive pressure sensors across various applications.

## 1. Introduction

With advancements in electronic control and materials science, flexible sensing technology has rapidly evolved, significantly enhancing the sensitivity and lightweight characteristics of sensors. This progress has led to innovative solutions across various fields, particularly in robotics [1], health monitoring [2], electronic skin [3], human–machine interfaces [4], and wearable devices [5]. In response to practical application needs, flexible sensors with diverse detection capabilities such as pressure, strain, gas, temperature, humidity, and light sensitivity have been developed [6]. Among these, flexible pressure sensors can be categorized into several types, including capacitive [7], resistive [8], piezoelectric [9], triboelectric [10], and emerging electrochemical sensors [11], based on their output signals or operational mechanisms. Notably, flexible capacitive pressure sensors offer several advantages, including a simple structure, rapid response time, straightforward signal acquisition, ease of large area manufacturing, and low energy consumption, making them particularly appealing [12].

Underwater thrusters operate on the principle that the movement of an external mechanical structure induces a change in pressure, facilitating water flow and, consequently, propulsion. The diaphragm is divided into two chambers. Driven by control signals and an electromagnetic field, the diaphragm moves vertically. By altering the volume of the upper and lower chambers, water is pumped and expelled. The pressure within the working chambers of thrusters and submersibles directly influences propulsion efficiency and diving operations. Traditional pressure sensors are typically large and time-consuming to install, which can significantly occupy internal space and increase structural complexity, leading to various challenges in detecting chamber pressure. In contrast, flexible sensors can effectively identify and measure chamber pressure in a straightforward and efficient manner, which is crucial for enhancing their practical application in monitoring underwater thruster movement [13]. Unlike scenarios involving wearable flexible sensors, the operational environment of underwater thrusters is complex, characterized by rapid changes in water pressure and significant fluctuations in amplitude. This necessitates that flexible pressure sensors possess high response speed and sensitivity.

**Figure 1:**
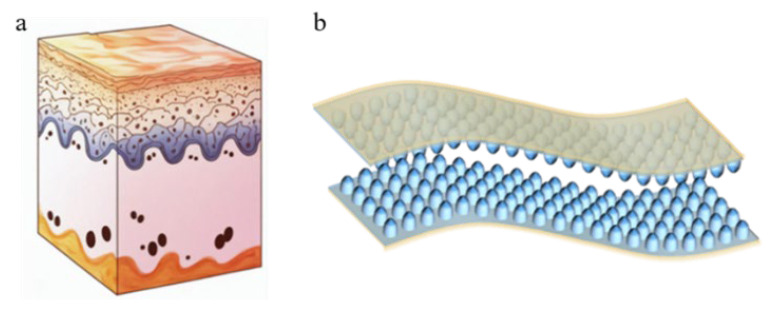
Skin papillary layer structure and sensor structure: (**a**) schematic diagram of the skin papilla structure; (**b**) schematic diagram of the sensor structure.

Human skin, with its complex microstructure, serves as an inspiration for the design of these sensors. The dermis layer, which contains nerve endings and blood vessels responsible for sensing touch, has a unique papillary layer structure, shown in Figure 1a, that can be used to improve the sensitivity of flexible pressure sensors [14].

Capacitive pressure sensors typically feature a “sandwich” structure, consisting of a dielectric layer positioned between two electrode layers. When external pressure is applied to the electrode layer, the distance between the two electrode plates decreases, resulting in a change in capacitance. The structure and composition of the dielectric layer determine its dielectric constant and directly influence the capacitance [15]. The sensitivity of capacitive pressure sensors can be improved in two ways. First, the dielectric layer microstructure can be constructed to make the dielectric layer of the sensor more prone to deformation, thus improving its sensitivity [16]. For example, the surface microstructures of natural organisms, such as lotus leaf [17], mimosa leaf [18], ginkgo leaf [19], indocalamus leaf [20], etc., can be used as templates. Alternatively, porous electrodes [21] or microstructure templates [22] can be prepared by photolithography [23], magnetron sputtering [24], etc. The microstructure pattern is transferred to the dielectric layer material by turning the mold. Common photolithographic or magnetron sputtering microstructures include microcylinder [25], pyramid [26], hemisphere [27], porous [28], pleated [29], conical [30], and sandpaper structures [31], which have been used to enhance the performance of flexible sensors [32]. However, due to the complex, time-consuming nature of the preparation process and the uneven and symmetrical microstructure, it is difficult to ensure consistency in the performance of a prepared batch of sensors [33]. Second, the material composition of the dielectric layer can be changed to increase its dielectric constant. The main materials that have been used include thermoplastic polyurethane (TPU) [34], MXene [35], hydrogels [36], and polydimethylsiloxane PDMS [37], which have good flexibility and low Young’s modulus and can be used in the preparation of nanoscale structures. PDMS offers the advantages of high elastic modulus, high permeability, and a simple fabrication process, making it suitable for the construction of miniaturized and highly integrated capacitive tactile sensors [38].

In this paper, we present a capacitive haptic sensor featuring a “Papillary” interlocking structure, as shown in Figure 1b, developed using a commercial nanoparticle anodized alumina (AAO) template [39,40,41] and polydimethylsiloxane (PDMS) as the dielectric layer material for reference by relevant scholars. The stability of the nanometer cylindrical ball head structure, combined with the excellent performance of PDMS, endows the tactile sensor with several advantages, including low pressure sensitivity, an ultralow detection limit, rapid response and recovery times, and exceptional stability during repeated loading and bending tests. The mechanical properties and capacitance characteristics of the pressure sensor were evaluated using a tensile testing machine and an LCR tester. The results demonstrate that the flexible sensor exhibits reliable capacitance characteristics under underwater operating conditions, enabling effective monitoring of chamber pressure.

## 2. Materials and Methods

### 2.1. Materials and Preparations

The PDMS precursor and curing agent are combined in a mass ratio of 10:1. The mixture is then subjected to vacuum treatment to eliminate any dissolved gases. Under the 20 °C and one standard atmosphere, the PDMS mixture is evenly poured onto the AAO diaphragm and heat-cured at 60 °C for 3 h. Once curing is complete, a cutter is used to trim the sample to external dimensions of 1 cm × 1 cm. The stacking process involves layering the electrodes, PDMS, and an additional electrode in a specified sequence. To ensure a strong bond between the layers, the sealing area around the chamber is coated with silicone rubber to maintain proper tightness.

**Figure 2:**
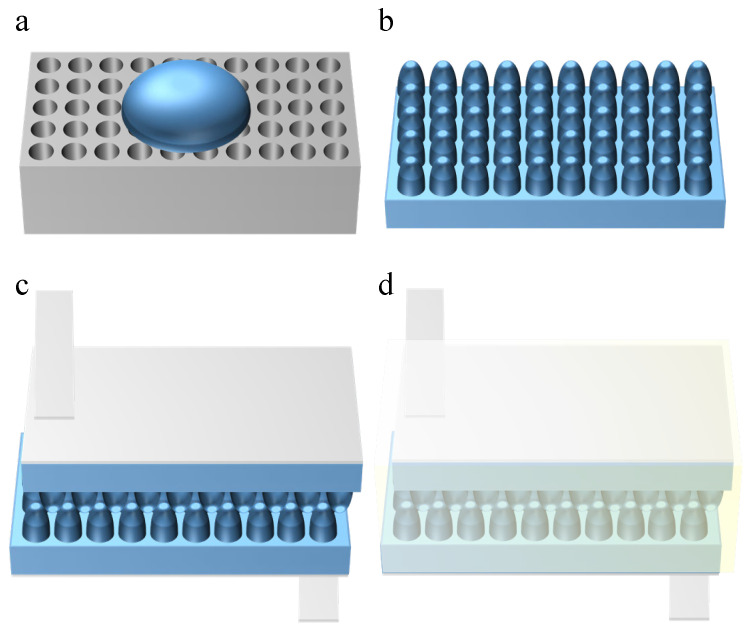
The manufacturing process of the sensor: (**a**) preparation of the PDMS dielectric layer; (**b**) schematic diagram of the dielectric layer structure; (**c**) preparation of the conductive electrode; (**d**) packaging of the sensor.

An AAO template with an aperture depth of 1000 nm is selected; this is cut into several square templates of 10 mm× 10 mm for use. If there are pollutants on the surface of AAO, it will directly affect the quality of dielectric layer preparation. The cut AAO template is put into ethanol solution and cleaned by an ultrasonic cleaning machine for 10 min; then, the ethanol solution is replaced, and the template is cleaned again by an ultrasonic cleaning machine for 10 min; finally, the template is soaked in deionized water for 20 min to remove any residual ethanol. The AAO template is removed from the deionized water and placed on a 70 °C baking table. A weight with a flat bottom is prepared, and this is pressed onto the AAO template to ensure smoothness after drying. The PDMS prepolymer and curing agent are mixed with a ratio of 10:1, and this is mechanically stirred for 10 min to ensure that a uniform gel liquid mixture is obtained. Then, this is left to stand for 40 min, removing the surface bubbles. The AAO template is put on the baking table; then, the configured PDMS mixture is poured onto the AAO template. The mixture is allowed to flow freely with gravity, as shown in Figure 2a. Then, the upper part of the AAO is covered with a thickness of 1 mm, and this is placed into a vacuum-drying oven for 20 min to ensure that the PDMS and AAO template are fully fitted and the surface is smooth. The AAO template and PDMS are placed on the pre-calibrated drying table at a temperature of 60 °C for 3 h to ensure the dielectric layer is cured. After the PDMS dielectric layer is cured, the film is slowly peeled off along the edge, and the AAO template and PDMS are washed with alcohol to obtain a dielectric layer structure that replicates the microstructure of the AAO template, as shown in Figure 2b. As shown in Figure 2c, the two PDMS dielectric layer microstructure surfaces are placed relative to each other, and the smooth surface is connected with conductive tape. As shown in Figure 2d, the sensor perimeter is sealed by silica gel, and finally the sensor is sealed again with insulation tape.

AAO templates: Nano-anodized aluminum oxide (AAO) templates with a pore diameter of 500 nm and pore depth of 1000 nm were purchased from Guangzhou (China) Hongwu Material Technology Co., Ltd.

PDMS: Sylgard 184 polydimethylsiloxane (PDMS) elastomer (Dow Corning).

Conductive tape: Conductive copper tape (0.1 mm thickness, 10 mm width) from Guangzhou (China) Penxing Technology Co., Ltd. was used for electrode connections.

Insulating tape: Polyimide insulating tape (20 mm width) was employed to ensure electrical isolation.

Silicone rubber: Room-temperature vulcanizing (RTV) silicone rubber (20 A hardness) from Shenzhen (China) Puston Silicone Material Co., Ltd. was applied for hermetic sealing.

### 2.2. Preparation and Characterization

**Figure 3:**
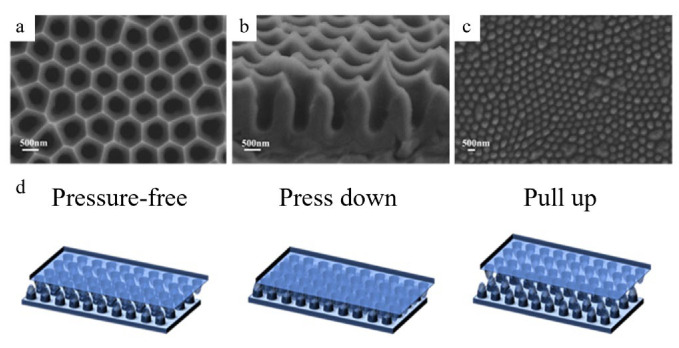
Preparation of the sensor papillary layer structure: (**a**) top view SEM image of the AAO template; (**b**) profile view SEM image of the AAO template; (**c**) top view SEM image of the PDMS nano-tentacle array; (**d**) schematic diagram of sensor working state.

Figure 3 presents a flow diagram illustrating the manufacturing process of the antiskid papillary bulge, along with a schematic representation of the final assembled capacitive pressure sensor. Additionally, we employed a cylindrical nanoparticle AAO template to create papillary bumps. By evenly distributing PDMS within the AAO template, it formed two nano-cylindrical arrays, which were subsequently arranged in an interlocking configuration. The capacitive tactile sensor was then assembled by applying ITO to both sides of the sensor. The completed PDMS and assembled sensors are depicted in Figure 3b. A cross-sectional scanning electron microscopy (SEM) image is also shown for an AAO template with a total depth of 1000 nm. These images reveal that there is no residue on the surface of the AAO template and that the array is well organized, indicating that the PDMS material successfully replicates the morphology of the AAO template.

## 3. Results

### 3.1. Principle and Performance Testing

**Figure 4:**
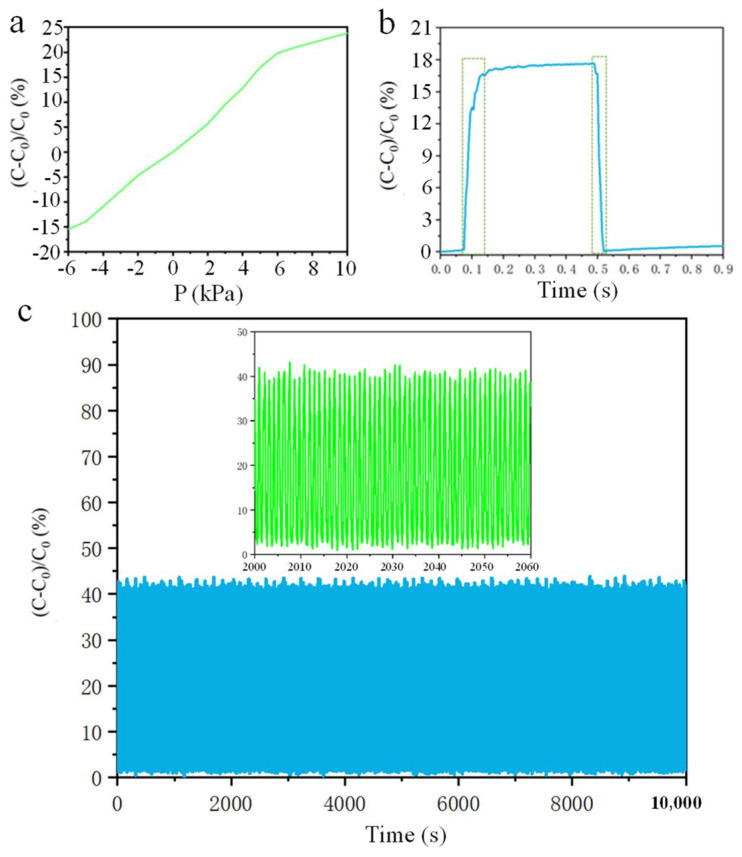
Sensor performance test: (**a**) sensor sensitivity test (Pressure-capacitance value change rate curve); (**b**) sensor response speed test (Time-capacitance value curve); (**c**) sensor durability test (Time-capacitance value curve).

The sensor described in this paper effectively measures both positive and negative pressures, and its sensing mechanism is outlined as follows: one side of the PDMS (polydimethylsiloxane) design is smooth, while the other side features a microstructure. The smooth side is fully in contact with the electrode, and the contact surface between the microstructure elements is the primary focus. For detecting negative pressure, when the pressure varies between 10 and −6 kPa, the capacitance value corresponds to the curve, as illustrated in Figure 4a. In the initial state, the surfaces of the microstructure are partially in contact with one another. When positive pressure is applied, the contact area between the electrode and the PDMS remains constant under downward force. As positive pressure increases, the PDMS begins to deform, resulting in an increased contact area between the PDMS microstructure and the electrode. Once the positive pressure reaches a certain threshold (about 6.3 kPa), the change in capacitance becomes less significant. Conversely, when the electrode experiences negative pressure, the contact area between the electrode and the PDMS begins to decrease under upward force in the chamber, leading to a more pronounced reduction in capacitance. As negative pressure increases, the contact area between the PDMS microstructure and the electrode gradually diminishes, and the sensor’s sensitivity also exhibits a decreasing trend at the certain pressure threshold (about 5.2 kPa).

The sensitivity of a capacitive tactile sensor is a key metric in evaluating its performance and is typically defined as the slope of the tangent line between the relative capacitance and applied pressure, which is measured at 3.27%/kPa in the range of −5.2 kPa to 6.3 kPa. As illustrated in Figure 3a, the real-time monitoring results indicate that the change in relative capacitance significantly increases with the application of vertical load pressure, demonstrating that the tactile sensor can maintain stable operating performance across a wide range of pressures.

In addition, we studied the response time of the proposed tactile sensor by applying weights to it, with the relevant results illustrated in Figure 4b. When an object is placed on the developed tactile sensor, the relative capacitance change increases significantly from the minimum to the maximum, with a unit time Δt1 of 52 ms. Conversely, when the object is removed from the sensor, the corresponding recovery time is Δt2 = 40 ms. These results indicate that the developed tactile sensor mimics human skin in terms of response and recovery time (30 ms, 60 ms). This exceptional performance can be attributed to the excellent elasticity and low viscosity properties of the micro-nanostructures modeled after skin. From this analysis, we conclude that the capacitive tactile sensor based on the skin mastoid structure exhibits high sensitivity under low pressure, although sensitivity gradually decreases as pressure increases. The stability test of the sensor is shown in Figure 4c. The sensor is fixed on the mouse and clicked 10,000 times.

We note that the proposed nano-cylindrical microstructures exhibit rapid response times and exceptional stability. Two key factors contribute to these remarkable characteristics: First, the uniform and dense distribution of the nano-cylindrical microstructure significantly reduces its viscoelasticity. The nano-cylinders can be evenly compressed under applied pressure and quickly return to their original state once the pressure is removed, resulting in very short response and recovery times. Secondly, the excellent stability of the nano-cylindrical microstructure can be attributed to the uniformity in the overall size of the nano-cylinders, which prevents the performance instability that could arise from discontinuities in similar conical structures. In summary, nano-cylindrical microstructures offer significant advantages for capacitive tactile sensors in terms of sensitivity, response and recovery times, and stability.

### 3.2. Detection of Human Motor Behavior

**Figure 5:**
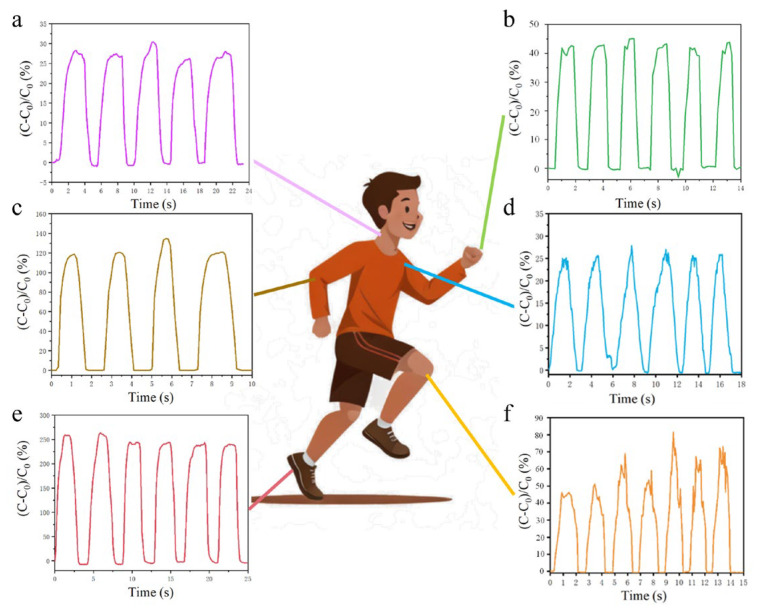
Monitoring of human movement behavior: (**a**) neck motion test; (**b**) finger flex test; (**c**) elbow flex test; (**d**) shoulder exercise test; (**e**) foot stress test; (**f**) knee bend test.

As shown in Figure 5a, the neck is bent from vertical to parallel to the ground, and then the capacitance time curve is lifted for a total of five cycles. Figure 5b shows the capacitance time characteristic curve of the sensor when the bent finger is held at 90° for a period of time and then extended. The sensor is mounted on the elbow joint, and the elbow-bending action is repeated to detect the change rule of the sensor, as shown in Figure 5c. As can be seen from the figure, the capacitance increases during elbow bending and reaches its highest point when the bending movement is completed. The prepared sensor was fixed at the joint of the shoulder and the upper arm of the hand, and the two ends of the sensor were connected with the LCR to test the capacitance time curve of lowering and raising the arm from the horizontal state for five cycles, as shown in Figure 5d. The sensor was fixed under the foot, and the test capacitance time characteristic curve of six consecutive cycles of high leg lifting was shown in Figure 5e for the tested person (weight 100 kg). Figure 5f shows the test signal of the human body during a squatting movement. People first squat slowly, then pause for a short time, and then rise slowly. The signal is stable within 7 cycles, and the capacitor signal is stable during squatting, which verifies the stability of the strain sensor on the strain detection performance.

## 4. Discussion

**Figure 6:**
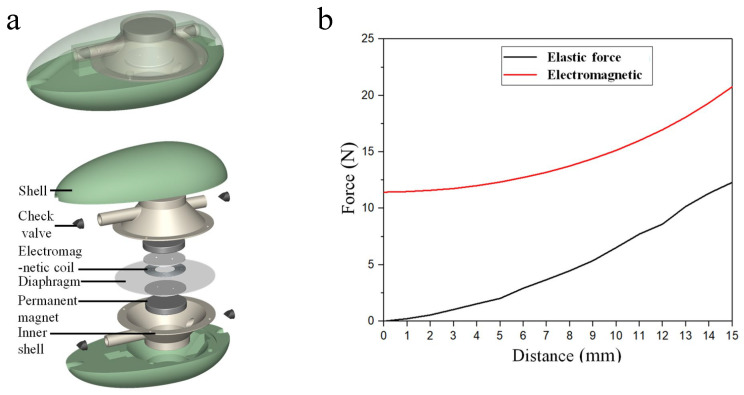
Pressure change of underwater thruster: (**a**) thruster structure; (**b**) electromagnetic force and diaphragm force.

Consider the biological function and the deformation mode of cuttlefish, as well as the resistance and energy consumption of cuttlefish in the process of suction and water intake; we believe that, if the unidirectional flow in the suction and drainage cycle can be realized, it can bring a better propulsion effect. A new type of suction and drainage propulsion is proposed, in which a water inlet is arranged at the front end of the thruster and a water outlet is arranged at the back end. The process of a water inlet at the front end and water spraying at the back end can produce a propulsion effect on the thruster, and the phenomenon of resistance and energy consumption caused by the water inlet process of the squid in the forward direction is eliminated. To meet the requirements of underwater continuous propulsion, we designed a thruster with an ellipsoidal shell, an upper and lower cone inner shell, a diaphragm separating the inner chamber, forming a double chamber, an electromagnetic force driving the diaphragm to move up and down, and an upper and lower chamber alternately spraying water, as shown in Figure 6a. The above is the overall effect diagram after translucent treatment, and the following is the explosion diagram of the main functional components of the thruster. It consists of two shells, two inner shells, two permanent magnets, one diaphragm, one electromagnetic coil, two baffles, and two bolts for ensuring that the electromagnetic coil and the diaphragm are fixed into one body. One end of each inner shell is a spray pipe, the other end is an inlet pipe, and a check valve is installed at the port of the pipe. Adopting the electromagnetic force drive mode, the electromagnetic coil and the silicone diaphragm constitute the whole movement, and the electromagnetic coil drives the silicone diaphragm up and down. Through the control of the direction of current, the magnetic field exerts upward force on the electromagnetic coil, and the electromagnetic coil drives the silicone diaphragm to move upward. The internal volume of the upper chamber decreases, and the water in the upper chamber flows to the left and is discharged out of the chamber. At the same time, the internal volume of the lower chamber increases, and the water outside the chamber flows into the chamber through the right check valve. By changing the direction of current, the magnetic field generates a downward driving force on the electromagnetic coil, and the electromagnetic coil drives the silicone diaphragm to move downward. When the internal volume of the upper chamber increases, the water outside the chamber flows into the chamber from the right side; when the internal volume of the lower chamber decreases, the water inside the chamber flows to the left side and drains to the outside of the chamber. By changing the current direction periodically, the process of double-chamber suction and water spraying alternates, and the cycle is carried out to realize the continuous forward propulsion of the double-chamber thruster [42].

When the electromagnetic coil is in its lowest position, it drives the silicone diaphragm upward. Before the silicone diaphragm returns to its flat state, both the electromagnetic force and the tension in the diaphragm act in the upward direction. The absolute values of these two forces combine to create a resultant force, which exerts pressure on the water in the upper chamber. Once the silicone diaphragm returns to its flat state, the electromagnetic force continues to act upward while the diaphragm tension acts downward. The net force exerted by the water in the upper chamber is downward when the electromagnetic coil drives the silicone diaphragm to move upward. The magnitude of the driving force can be obtained by subtracting the absolute value of the diaphragm tension from that of the electromagnetic force.

When the electromagnetic coil is in its highest position, it drives the silicone diaphragm downward. Before the silicone diaphragm returns to a flat state, both the electromagnetic force and the tension in the diaphragm act downward. The absolute values of these two forces combine to create a resultant force, which exerts pressure on the water in the lower chamber. Once the silicone diaphragm returns to a flat state, the electromagnetic force continues to act downward while the diaphragm tension acts upward. The net force on the water in the lower chamber is upward when the electromagnetic coil drives the silicone diaphragm downward. The absolute value of the electromagnetic force can be derived through the subtraction of the absolute value of the diaphragm’s tension from the absolute value of the electromagnetic force. The magnitude of the driving force can be obtained by subtracting the absolute value of the diaphragm tension from that of the electromagnetic force. This analysis indicates that the water pressure in the upper chamber is theoretically negative when the diaphragm moves downward and theoretically positive when the diaphragm moves upward [43].

Polynomial fitting is used to fit the electromagnetic force at a current of 10 A $F_{\mathrm{mag}}$ shown in Figure 6. The polynomial function is expressed as

(1) $$F_{\mathrm{mag}}=49975500y^{4}+29669y^{2}+11.57$$

The polynomial function of the diaphragm elastic force $F_{\mathrm{ela}}$ gives

(2) $$F_{\mathrm{ela}}=-2464480000y^{5}+2515870y^{3}+543.24y$$

During the upward motion of the diaphragm, the pressure in the upper chamber increases, which causes the water in the upper chamber to jet outwards. At the same time, the pressure in the lower chamber decreases and the lower chamber absorbs water from the outside. In the process of the downward motion of the diaphragm, the pressure in the lower chamber increases and the water in the lower chamber jets outward, while the pressure in the upper chamber decreases and the upper chamber absorbs water from the outside. It is important to note that the overall volume of water contained within both chambers remains constant throughout this process. The total mass of the moving combination is m=0.177 kg, and the mass of the diaphragm assembly is $m_{\mathrm{coil}}=0.04\;\mathrm{kg}$. The gravitational force of the moving combination including the water in the chamber and the diaphragm assembly is

(3) G=mg=1.77 N

The solution to water pressure in the upper chamber involves calculating the current dynamic volume of the upper chamber, the flow rate of the outlet, and the outlet pressure. Combined with the data presented in Table 1, the current dynamic volume of the upper chamber without the tubes is expressed as

(4) $$\begin{array}{l} V_{\mathrm{upcham}}=\frac{\pi }{3}(R_{\mathrm{dia}}^{2}+r_{\mathrm{mag}}^{2}+R_{\mathrm{dia}}\text{×}r_{\mathrm{mag}})(h-y)\\ =0.0046(0.015-y) \end{array}$$

where $R_{\mathrm{dia}}$ represents the inner radius of the large end of the chamber, which is equal to the radius of the diaphragm’s motion area. $r_{\mathrm{mag}}$ represents the inner radius of the small end of the chamber, which is equal to the radius of the magnet. h is the maximum displacement on one side of the diaphragm. As the y value increases, the volume of the upper chamber gradually decreases, causing water to jet outward from the outlet. The derivative of volume with respect to time gives the flow rate of the outlet in the upper chamber.

(5) $$q_{\mathrm{outlet}}=\frac{dV_{\mathrm{upcham}}}{dt}=-0.0046\dot{y}$$

The flow velocity of the water jet at the outlet is given by

(6) $$v_{\mathrm{outlet}}=\frac{q_{\mathrm{outlet}}}{S_{\mathrm{outlet}}}=-58.3\dot{y}$$

where $S_{\mathrm{outlet}}$ represents the area of the outlet. Considering the pressure loss combined with the outlet flow rate, the propulsion of the outlet in the upper chamber can be determined.

(7) $$F_{\mathrm{out}}=F\prime_{\mathrm{out}}=\frac{1}{2}\times q_{\mathrm{outlet}}\times \rho \times v_{\mathrm{outlet}}=133.6{\dot{y}}^{2}$$

The derivative of velocity with respect to time yields the current acceleration of water flow in the outlet tube.

(8) $$a_{\mathrm{outlet}}={\dot{v}}_{\mathrm{outlet}}=-58.3\ddot{y}$$

The static pressure at the outlet is equal to the external water, and the dynamic pressure of the water at the outlet is given by

(9) $$p_{\mathrm{outlet}}=\frac{1}{2}\rho v_{\mathrm{outlet}}^{2}$$

According to Bernoulli’s equation:

(10) $$\frac{p_{\mathrm{upcham}}}{\rho g}=\frac{v_{\mathrm{outlet}}^{2}}{2g}-\frac{{\dot{y}}^{2}}{2g}$$

The section area of the upper chamber is much larger than the section area of the water outlet, $\dot{y}\ll v_{\mathrm{outlet}}$. According to the above formula, we can obtain

(11) $$p_{\mathrm{upcham}}=\frac{1}{2}\rho v_{\mathrm{outlet}}^{2}$$

(12) $$p_{\mathrm{upcham}}=1701230{\dot{y}}^{2}$$

where $F_{\mathrm{up}}$ is the pressure of the pressure sensor in the upper chamber equal to the internal water pressure multiplied by the area of the pressure sensor and S is the area of the pressure sensor in the inner chamber.

(13) $$F_{\mathrm{up}}=p_{\mathrm{upcham}}S=170.123{\dot{y}}^{2}$$

**Figure 7:**
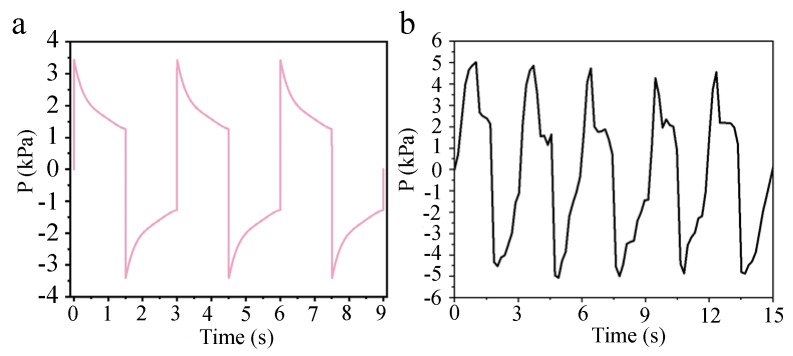
Pressure change of underwater thruster: (**a**) theoretical pressure change curve; (**b**) time–pressure change curve.

Figure 7a illustrates the process of change in the theoretical pressure test values of the pressure sensor located within the upper chamber. To effectively monitor the motion state of the thruster and the pressure variations within the propulsion device, we set the driving current of the electromagnetic coil to 10 A and conducted a 2-h underwater propulsion test. During this test, we recorded the sensor capacitance signal over a duration of 18 s, capturing six movement cycles. As depicted in Figure 7b, the capacitance of the pressure sensor exhibits a periodic pattern. The data indicate that the pressure measured by the sensor fluctuates between positive and negative values, suggesting that the water pressure within the upper chamber alternates between positive and negative, with water flowing in and out from the external environment. This observation confirms the practicality of the underwater pressure sensor and its capability to detect both positive and negative pressure. The periodicity detected by the sensor aligns with the values obtained through calculations, and the overall trend of pressure change is consistent.

## 5. Conclusions

The purpose of this paper is to develop a compact, rapid-response pressure sensor for underwater propulsion. Inspired by the interlocking micro-ridge structure found between the dermis and epidermis of human skin, we have devised a straightforward method for creating transfer techniques to prepare a capacitive tactile sensor, featuring a highly sensitive cylindrical Wiener structure. The dielectric layer of the capacitive pressure sensor is composed of polydimethylsiloxane (PDMS), and silicone rubber is utilized as an adhesive to maintain a sealed environment. The sensing mechanism is based on the change in the contact area between the hydrogel and the electrode under the internal and external pressure difference, which leads to a change in capacitance, so that the positive and negative pressure can be measured. The experimental results show that the uniform and densely distributed cylindrical microstructure can highly concentrate the stress, thus achieving the high performance of the proposed tactile sensor. The capacitive sensor shows a good linear rule in the range of −5.2 kPa to 6.3 kPa, and the sensor had a quick response time of 52 ms, a recovery time of 40 ms, and excellent stability. The pressure of the capacitive sensor for pressure detection inside the underwater thruster changes back and forth between positive and negative, indicating that there are positive and negative water pressures inside the upper chamber; accordingly, the thruster can achieve suction and drainage and thus can achieve propulsion function, which also verifies the practicality of the underwater pressure sensor and the function of detecting positive and negative pressures.

## Figures and Tables

**Table 1:** Main dimensions of thruster.

Components	Parameter	Value	Unit
Shell	Max. length Max. width Max. height Thickness of wall Inlet radius Outlet radius Outlet radius Outlet length	200 120 45 2 5 5 5 55	mm mm mm mm mm mm mm mm
Inner shell	Inlet radius Inlet length Chamber height Chamber bottom radius Chamber top surface radius	5 30 25 50 25	mm mm mm mm mm
Electromagnetic coil	outer diameter inner diameter wire diameter turns Radius	50 35 0.4 150 25	mm mm mm turn mm
Permanent magnet	Height	10	mm
Overall	Weight	562.3	g

## Data Availability

Data are contained within the article.
